# Rehabilitation in ICU-Acquired Weakness: An Evidence-Based Narrative Review

**DOI:** 10.7759/cureus.107933

**Published:** 2026-04-29

**Authors:** Sarika Mishra, Anurag Tripathi, Yesh Veer Singh, Ved Prakash, Virinder Singh Gogia, Hemant Kumar, Atul Tiwari, Mohammad Arif, Sachin Kumar, Sandeep Bhadu, Deepak Sharma

**Affiliations:** 1 Physical Medicine and Rehabilitation, Dr. Ram Manohar Lohia Institute of Medical Sciences, Lucknow, IND; 2 Pulmonary and Critical Care Medicine, King George's Medical University, Lucknow, IND; 3 Respiratory Medicine, Dr. Ram Manohar Lohia Institute of Medical Sciences, Lucknow, IND; 4 Respiratory Medicine, Institute of Medical Sciences, Varanasi, IND; 5 Pulmonary and Critical Care, King George's Medical University, Lucknow, IND; 6 Pulmonary Medicine, Shanti Devi GI Institute, Hisar, IND

**Keywords:** critical illness myopathy, critical illness polyneuropathy, intensive care unit-acquired weakness (icu-aw), mechanical ventilation (mv), physical therapy rehabilitation

## Abstract

Neuromuscular weakness due to critical illness myopathy and polyneuropathy is a common and disabling complication of modern intensive care, contributing to prolonged ventilator dependence, delayed recovery, and persistent functional impairment among ICU survivors. With improving survival from critical illness - particularly in low- and middle-income countries - the burden of ICU-acquired weakness has increased, while rehabilitation practices remain heterogeneous and inconsistently implemented. This narrative review, developed in accordance with the Scale for the Assessment of Narrative Review Articles (SANRA) criteria, synthesises contemporary evidence on rehabilitation strategies for ICU-acquired neuromuscular weakness using a structured, non-systematic search of major databases covering publications from January 1980 to January 2025.

Current evidence suggests that structured, multidisciplinary rehabilitation improves short-term muscle strength, mobility, and healthcare efficiency, although consistent benefits on mortality and long-term quality of life have not been demonstrated. Early active mobilisation increases activity levels and attainment of functional milestones but requires individualisation to ensure safety and effectiveness. Adjunctive modalities such as neuromuscular electrical stimulation and inspiratory muscle training show benefit in selected patients, particularly when integrated with conventional physiotherapy, whereas passive interventions predominantly appear insufficient when used in isolation. Diagnostic and prognostic assessment should prioritise early identification of clinically relevant weakness to enable timely rehabilitation rather than exhaustive neuromuscular classification.

This review integrates key pathophysiological mechanisms, pragmatic diagnostic approaches, evidence-based rehabilitation modalities, and real-world barriers to implementation. Rehabilitation in ICU-acquired neuromuscular weakness should be regarded as a core therapeutic intervention - initiated early when feasible, titrated to illness severity, and integrated across the ICU-to-recovery continuum - to optimise outcomes for survivors of critical illness.

## Introduction and background

Neuromuscular weakness is a frequent and clinically important complication of modern intensive care, reflecting improved survival from severe sepsis, multiorgan failure, and prolonged critical illness, as well as exposure to sedative, corticosteroid, and neuromuscular-blocking therapies [1]. In critically ill adults, this weakness most commonly arises from acquired dysfunction of skeletal muscle and peripheral nerves, classically described as critical illness myopathy (CIM) and critical illness polyneuropathy (CIP). In routine clinical practice, however, isolated phenotypes are uncommon, and simultaneous involvement of muscle and nerve predominates, often precluding clear phenotypic separation [1]. Consequently, broader umbrella terms, including CIM and CIP, are increasingly used to describe this continuum of ICU-acquired neuromuscular dysfunction [2,3].

ICU-acquired neuromuscular weakness affects approximately one-quarter to two-thirds of patients requiring prolonged mechanical ventilation, with prevalence approaching 60-70% in patients with severe sepsis and prolonged critical illness [1,2,4,5]. The condition predominantly affects adults, particularly older individuals, while paediatric cases remain relatively uncommon [6]. Importantly, its clinical relevance extends beyond reduced limb strength. ICU-acquired weakness (ICU-AW) is independently associated with prolonged ventilator dependence, delayed liberation from intensive care, increased healthcare utilisation, and persistent functional disability after hospital discharge, and has been linked to increased short- and long-term mortality [7-9]. ICU-AW also represents a key physical component of Post-Intensive Care Syndrome (PICS), contributing significantly to long-term functional impairment among ICU survivors.

Despite the growing recognition of its substantial impact on survival and long-term recovery, rehabilitation practices targeting ICU-acquired neuromuscular weakness remain highly variable across centres. Key uncertainties persist regarding the optimal timing, intensity, and composition of rehabilitation interventions, particularly in patients with established CIM or CIP. Available evidence is characterised by methodological heterogeneity and inconsistent outcome reporting, limiting translation into routine clinical practice.

Given the absence of disease-specific pharmacological therapies for critical illness-associated neuromyopathy and the expanding population of ICU survivors, rehabilitation has emerged as a central and potentially modifiable component of care. Recent large trials and meta-analyses have refined the understanding of early mobilisation and adjunctive rehabilitation strategies, while highlighting important limitations of uniform approaches [10,11].

Early rehabilitation broadly refers to the timely initiation of graded physical and functional interventions during critical illness, tailored to patient stability and recovery trajectory. A focused synthesis of current rehabilitation strategies is therefore warranted to inform bedside decision-making, delineate critical evidence gaps, and guide future research and guideline development.

## Review

Methods

This narrative review was conducted in accordance with the Scale for the Assessment of Narrative Review Articles (SANRA) criteria [12]. The manuscript was evaluated using the SANRA checklist, achieving a total score of 10/12, indicating high methodological quality (Appendix). A structured, non-systematic literature search was performed using PubMed/MEDLINE, Embase, the Cochrane Library, and Google Scholar for publications from January 1980 to January 2025, encompassing both foundational descriptions and contemporary rehabilitation evidence.

The search strategy included combinations of keywords and Medical Subject Headings (MeSH) such as “ICU-acquired weakness,” “critical illness myopathy,” “critical illness polyneuropathy,” “rehabilitation,” “early mobilisation,” “physiotherapy,” “neuromuscular electrical stimulation,” and “respiratory muscle training,” combined using Boolean operators (e.g., “ICU-acquired weakness AND rehabilitation,” “early mobilisation AND critical illness”). Paediatric studies, non-peer-reviewed articles, and non-English publications were excluded. Reference lists of key articles were screened to identify additional relevant studies.

An example of the search strategy used in PubMed was as follows: (‘ICU-acquired weakness’ OR ‘critical illness myopathy’ OR ‘critical illness polyneuropathy’) AND (‘rehabilitation’ OR ‘early mobilisation’ OR ‘physiotherapy’ OR ‘neuromuscular electrical stimulation’ OR ‘respiratory muscle training’). This search yielded approximately 500 records, of which around 370 were screened after duplicate removal. Approximately 120-140 full-text articles were assessed for eligibility, and about 70 studies were included in the narrative synthesis based on relevance to predefined thematic domains.

Evidence was synthesised narratively and thematically across domains of pathophysiology, diagnosis, rehabilitation strategies, clinical outcomes, and implementation barriers. The synthesis was qualitative. Formal risk-of-bias assessment and meta-analysis were not undertaken due to heterogeneity of evidence.

Impact of ICU-AW

Clinical assessment at the time of recovery of consciousness consistently demonstrates that patients who develop ICU-AW experience higher ICU and in-hospital mortality compared with those without neuromuscular involvement [7,8]. This association appears most robust for weakness affecting the limb musculature [9], whereas the prognostic significance of isolated diaphragmatic dysfunction, although increasingly recognised, has shown greater variability across studies [9,13].

Both peripheral and respiratory muscle weakness independently contribute to prolonged dependence on mechanical ventilation and delayed liberation from ventilatory support [14,15]. In medical ICU populations, limb muscle weakness identified at the time of planned extubation has been associated with an increased risk of extubation failure [16]. In contrast, studies involving predominantly surgical cohorts suggest that combined limb and diaphragmatic dysfunction is common and confers particularly poor outcomes, with approximately half of affected patients requiring re-intubation within 72 hours and experiencing substantial subsequent mortality [17].

The impact of ICU-AW extends beyond respiratory failure. Affected patients frequently have longer ICU and hospital stays and incur higher healthcare costs [9,18,19]. Neuromuscular dysfunction has also been linked to non-respiratory complications, including post-extubation dysphagia [20] and impaired cough effectiveness due to abdominal muscle weakness, which may further increase the risk of pulmonary complications and delay recovery [21].

Pathophysiology

Prolonged immobility is a central driver of neuromuscular dysfunction during critical illness. Reduced mechanical loading leads to rapid structural and functional changes in skeletal muscle, resulting in disuse atrophy, joint contractures, metabolic derangements, and secondary complications such as thromboembolism and pressure-related injury [22]. Imaging and biopsy studies demonstrate accelerated muscle wasting early during ICU stay, with substantial cumulative loss occurring within the first two weeks of immobilisation [23,24]. Severe muscle loss is associated with poorer clinical outcomes, including increased mortality.

At the cellular level, muscle wasting reflects reduced muscle fibre cross-sectional area - particularly in antigravity muscles - and activation of catabolic pathways that impair force generation [25]. In CIM, preferential degradation of myosin contributes to early and profound weakness, which may progress rapidly during sustained bed rest or limb immobilisation [22,25,26].

Systemic inflammation associated with sepsis and multiorgan failure further exacerbates neuromuscular injury through metabolic, microvascular, and bioenergetic disturbances affecting both skeletal muscle and peripheral nerves [25]. Concurrent exposure to deep sedation, neuromuscular blockade, and controlled mechanical ventilation suppresses spontaneous muscle activation (“mechanical silencing”), accelerating limb and diaphragmatic atrophy and contributing to ventilator dependence [27].

Pharmacological factors compound these effects. Neuromuscular blocking agents impair neuromuscular transmission and may potentiate corticosteroid-associated myotoxicity [28], while sedative agents suppress voluntary and reflex muscle activity, reinforcing immobility-related muscle loss [25]. Although inadequate nutrition worsens catabolism, early caloric and protein supplementation alone has not reliably prevented muscle wasting, underscoring the multifactorial pathogenesis of ICU-acquired neuromyopathy [27].

**Figure 1:**
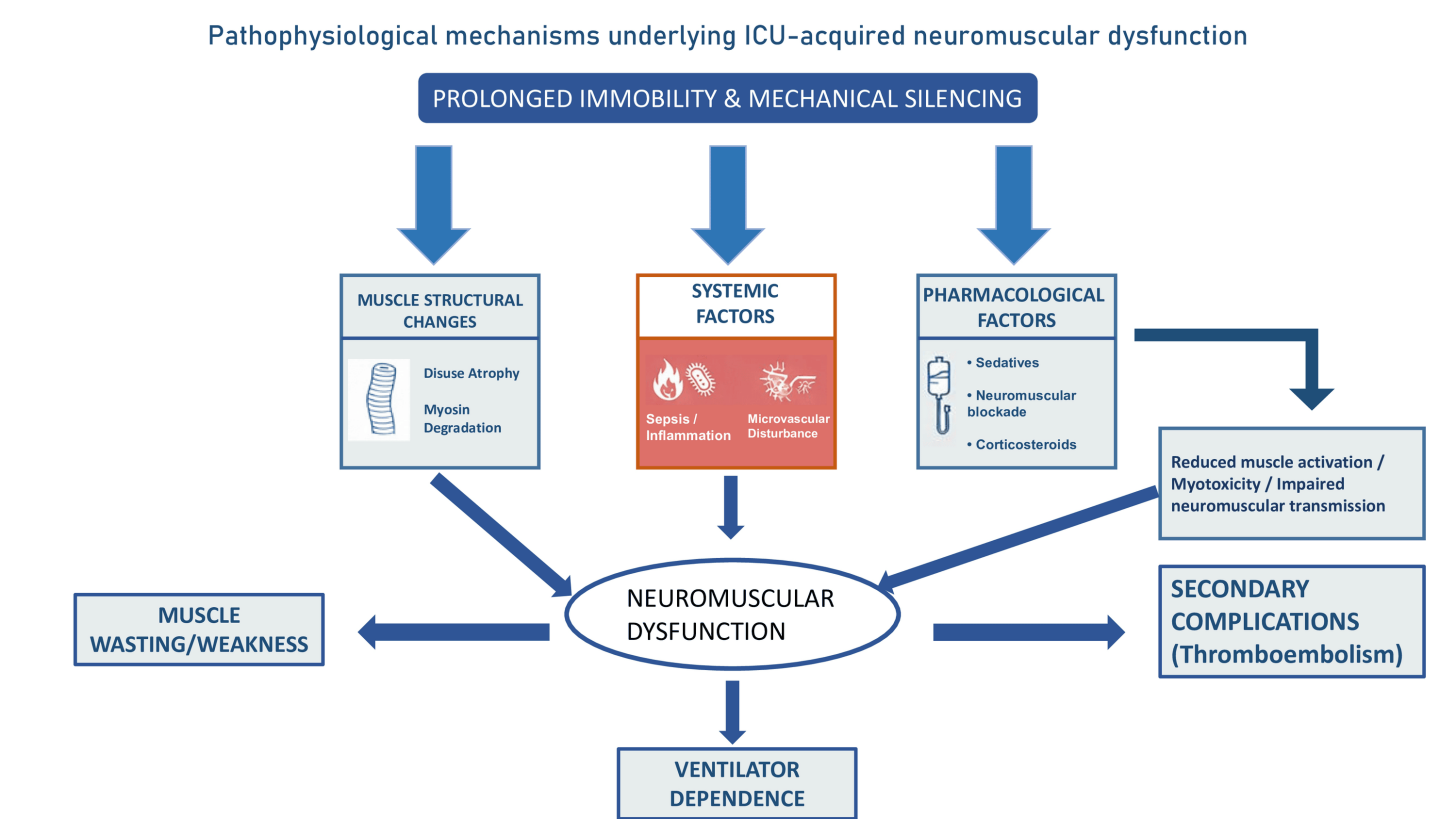
Pathophysiological mechanisms underlying ICU-acquired neuromuscular dysfunction This figure illustrates the interaction between immobilisation, systemic inflammation, pharmacological exposures, and metabolic dysfunction leading to neuromuscular injury and clinical complications [22,25,27,28]. Image credits: Created by Dr. Anurag Tripathi, using Microsoft PowerPoint (Microsoft Corp., Redmond, WA, USA).

The pathophysiology of ICU-AW is summarised in Figure 1.

Clinical features & diagnosis

CIP and CIM frequently coexist and are best approached clinically under the umbrella of ICU-AW rather than as strictly separate entities. For the intensivist, the key diagnostic question is not precise phenotyping, but early recognition of clinically relevant weakness that may delay ventilator liberation and functional recovery.

The diagnostic approach is therefore guided primarily by patient cooperation and illness severity. In alert and cooperative patients, bedside assessment of global muscle strength remains the cornerstone of diagnosis. The Medical Research Council (MRC) sum score provides a simple and reproducible method to quantify limb weakness, with established thresholds identifying clinically significant and severe ICU-AW [1,29]. When formal manual testing is not feasible, handgrip dynamometry offers a pragmatic screening tool and has been shown to correlate with global strength and clinical outcomes [30,31].

In patients who are deeply sedated, delirious, or unable to participate in voluntary testing, electrophysiological evaluation plays a supportive role. Simplified nerve conduction studies can identify early neuromuscular dysfunction before overt weakness becomes apparent and may assist in prognostication [7]. While electrophysiology can help distinguish predominantly neuropathic from myopathic patterns, such differentiation rarely alters immediate ICU management. Importantly, electrophysiological abnormalities have been associated with prolonged ventilator dependence, impaired functional recovery, and increased long-term mortality, reinforcing their value as markers of disease severity rather than as purely diagnostic tools [32-34].

From a practical standpoint, the diagnosis of ICU-AW should be viewed as a clinical trigger for early, structured rehabilitation and optimisation of modifiable risk factors, rather than as a requirement for exhaustive neuromuscular classification.

A pragmatic, ICU-focused diagnostic approach is summarised in Table 1.

**Table 1:** Practical diagnostic approach to ICU-acquired weakness ICU-AW: ICU-acquired weakness; CMAP: Compound Muscle Action Potential.

Clinical situation	Tool	What it tells you	Key limitation
Awake, cooperative patient	Medical Research Council (MRC) sum score	Quantifies global limb weakness; identifies clinically significant ICU-AW	Requires patient cooperation; does not localise pathology
Awake but fatigued or partially cooperative	Handgrip dynamometry	Rapid screening for reduced muscle strength; prognostic value	Influenced by effort and cognition
Sedated or uncooperative patient	Simplified nerve conduction studies (e.g. peroneal CMAP); Muscle Ultrasound	Early detection of neuromuscular dysfunction: prognostic information Non-invasive bedside assessment of muscle mass and architecture; enables early detection and longitudinal monitoring of muscle wasting.	Limited availability; requires expertise; Operator dependent; variability in measurement techniques; limited ability to distinguish myopathy from neuropathy.
Suspected myopathy vs neuropathy	Electromyography ± direct muscle stimulation	Differentiates myopathic from neuropathic patterns	Rarely changes acute ICU management
Persistent weakness after ICU discharge	Comprehensive electrophysiology ± imaging	Characterises residual neuromuscular injury	Limited relevance for early ICU rehabilitation decisions

Rationale for rehabilitation in CIM and CIP

Rehabilitation interventions in CIM and CIP aim to mitigate the profound neuromuscular consequences of immobilisation and systemic inflammation. Early application of mechanical loading and neuromuscular activation may attenuate catabolic signalling, preserve muscle architecture, and maintain joint mobility, thereby supporting functional recovery. When voluntary exercise is not feasible, passive and assisted movements can help maintain range of motion, limit contracture formation, and promote local circulation, serving as a bridge to more active rehabilitation.

Rehabilitation also plays a pivotal role in respiratory recovery. Diaphragmatic atrophy and inspiratory muscle weakness are central contributors to weaning difficulty and prolonged ventilator dependence in CIM and CIP. Interventions targeting respiratory muscle performance, combined with early mobilisation, may improve ventilatory efficiency, enhance cough effectiveness, and facilitate liberation from mechanical ventilation.

Given this biological and clinical rationale, a staged rehabilitation approach tailored to illness severity and physiological stability is essential. ICU-based rehabilitation encompasses a continuum ranging from passive interventions in deeply sedated patients to progressively active, task-oriented exercises as consciousness, strength, and cardiorespiratory reserve improve. Each modality targets distinct neuromuscular and functional impairments encountered across the trajectory of critical illness [22,25].

Passive mobilisation

As consciousness and physiological stability improve, active-assisted and active mobilisation become central components of ICU rehabilitation. These interventions directly promote muscle activation, postural control, and functional retraining.

Moderate-certainty evidence indicates that early active mobilisation increases patient activity levels and accelerates attainment of short-term functional milestones. However, its effect on longer-term outcomes is inconsistent. In the multicentre Treatment of Mechanically Ventilated Adults with Early Activity and Mobilisation (TEAM) trial, early active mobilisation resulted in greater daily activity and earlier achievement of standing. Still, it did not improve days alive and out of the hospital at 180 days, mortality, functional status, quality of life, or cognitive outcomes. Mobilisation-related adverse events were more frequent in the intervention group, underscoring the need for individualised, safety-guided mobilisation rather than uniform protocols [35].

Overall, active mobilisation is most beneficial when progressively titrated to patient tolerance and integrated within multidisciplinary ICU care, rather than applied aggressively or indiscriminately. As patients regain consciousness and physiological stability, rehabilitation progresses from passive interventions to active and task-oriented mobilisation [36].

Active mobilisation

As patients regain consciousness and physiological stability, assisted active and active mobilisation become increasingly important. Active-assisted range-of-motion exercises allow patients to initiate movement with external support, facilitating muscle activation while accommodating residual weakness and fatigue. Progression to fully active movements promotes strengthening, joint mobility, and functional retraining through repeated, controlled activity.

Although early active mobilisation reliably increases patient activity levels and accelerates short-term functional milestones, its effect on longer-term outcomes remains variable, as seen in a large multicentre trial [35].

Bed mobility and sitting

Bed mobility and assisted sitting represent transitional steps between passive interventions and upright mobilisation. Although not evaluated as isolated interventions in randomised trials, moderate-certainty evidence from multimodal rehabilitation programmes supports their inclusion as part of structured early mobilisation strategies [37,38].

Systematic reviews indicate that programmes incorporating in-bed mobility, sitting, standing, and ambulation are associated with reduced ICU-acquired weakness, improved functional mobility, increased ventilator-free days, and higher likelihood of discharge to home, compared with usual care [35]. These findings support early progression from bed-based activities toward upright mobilisation within a staged rehabilitation framework.

Cycle ergometry

Bedside cycle ergometry enables repetitive lower-limb movement in immobile or sedated patients and can be progressed from passive to active-assisted or resistive modes as recovery allows. Low- to moderate-certainty evidence from early single-centre trials suggested potential benefits, including improved walking distance and physical function at hospital discharge and reduced prevalence of ICU-AW at ICU discharge [39]. However, these studies were limited by small sample sizes and heterogeneous protocols.

More robust evidence from a large multicentre randomised trial demonstrated that early in-bed cycling increased lower-limb activity but did not improve short-term physical function or reduce ICU-AW when added to standard physiotherapy [40]. The predominantly passive nature of cycling in this trial may have limited physiological benefit.

Overall, evidence remains inconsistent, and predominantly passive cycle ergometry appears unlikely to confer meaningful clinical benefit when used in isolation. It may retain value as part of a multimodal rehabilitation programme, particularly in patients unable to engage in active exercise during early critical illness [41].

Neuromuscular electrical stimulation (NMES)

NMES provides non-volitional muscle activation during periods of immobilisation and is particularly relevant for patients unable to participate in active exercise [42-44]. Evidence evaluating NMES is heterogeneous but promising. Low- to moderate-certainty evidence suggests that NMES can attenuate muscle wasting and may reduce the incidence of ICU-AW, particularly when combined with conventional physiotherapy.

In a randomised trial, ICU-AW occurred in none of the patients receiving combined NMES and range-of-motion exercises, compared with substantially higher rates in comparator groups [45]. Systematic reviews and network meta-analyses have reported improved extubation success with NMES, especially when delivered alongside active rehabilitation, but there were no consistent effects on length of stay, duration of mechanical ventilation, or mortality, with overall certainty rated as low to very low [46,47]. Collectively, current evidence supports NMES as an adjunctive intervention, rather than a replacement for active rehabilitation.

Respiratory muscle training

Respiratory muscle dysfunction, particularly diaphragmatic weakness, is a major contributor to weaning failure during critical illness [44,48]. Moderate-certainty evidence supports inspiratory muscle training (IMT) as a targeted intervention to improve inspiratory muscle strength and facilitate ventilator liberation in selected patients. Randomised trials and meta-analyses demonstrate improved inspiratory pressures and shorter weaning duration, particularly in patients with difficult or prolonged weaning [49-51]. However, consistent benefits on ICU length of stay or mortality have not been demonstrated.

Respiratory muscle training should therefore be considered a physiologically targeted adjunct, best applied in patients with demonstrable respiratory muscle weakness and integrated within broader rehabilitation strategies.

Early mobilisation protocols

Patient Selection and Safety

Safe early mobilisation in the ICU requires dynamic clinical judgement rather than rigid eligibility checklists. While absolute contraindications, such as acute myocardial infarction, uncontrolled intracranial pressure, active bleeding, or unstable spinal and pelvic fractures, preclude mobilisation, the majority of critically ill patients fall into a grey zone where risk-benefit assessment must be individualised [52]. In hemodynamically stable patients without escalating vasopressor requirements, mobilisation can often be safely initiated despite ongoing organ support, including non-invasive ventilation or low levels of invasive mechanical ventilation, provided respiratory parameters remain stable [10].

Practical screening focuses on four domains: cardiovascular stability, respiratory reserve, neurological responsiveness, and overall physiological resilience. Importantly, complete alertness is not a prerequisite for mobilisation. Selected patients with limited responsiveness may tolerate graded activity when closely monitored, challenging the traditional notion that mobilisation must wait for full awakening [52,53]. Similarly, moderate ventilator settings and the absence of severe patient-ventilator asynchrony are generally sufficient to permit mobilisation, rather than serving as absolute barriers [54].

Routine daily screening is central to successful mobilisation programmes, allowing therapy intensity to be adjusted in parallel with evolving illness severity and recovery trajectory [55]. However, evidence to date does not define a single “optimal” mobilisation threshold or timeline. This uncertainty underscores the importance of adaptive, patient-specific rehabilitation strategies rather than protocol-driven escalation of activity [10,56].

Despite consistent improvements in activity levels and short-term functional milestones, early mobilisation has not uniformly translated into better long-term outcomes, as highlighted by the neutral results (i.e., no significant improvement in median number of days patient alive and out of hospital and deaths by day 180) in large mobilisation trials [35].

Several factors likely contribute to this discrepancy. First, a mismatch between activity and physiological stimulus may occur. Increased activity does not necessarily provide sufficient mechanical loading to induce sustained muscle adaptation, particularly in patients with severe catabolism and systemic inflammation.

Second, patient heterogeneity plays a major role. Uniform mobilisation protocols applied across patients with varying illness severity, physiological reserve, and recovery potential may dilute measurable benefits.

Third, the timing of intervention relative to disease biology is critical. Mobilisation during phases of profound inflammation, metabolic dysfunction, and anabolic resistance may limit muscle responsiveness to exercise.

Fourth, long-term outcomes such as quality of life and functional independence are influenced by multiple non-musculoskeletal factors, including cognitive impairment, comorbidities, and post-ICU complications, reducing the isolated impact of mobilisation.

Finally, safety-driven limitations on exercise intensity are necessary to avoid adverse events but may constrain the physiological stimulus required for meaningful long-term adaptation.

These considerations highlight that early mobilisation should be viewed as a foundational component of ICU care that requires integration with targeted strengthening, respiratory muscle training, nutritional support, and post-ICU rehabilitation to influence long-term recovery [35,24].

Patient Cooperation and Cognitive Status

Patient engagement substantially influences the safety and effectiveness of mobilisation. Structured assessment of pain, agitation, sedation depth, delirium, and cooperation should precede therapy sessions [52]. Uncontrolled pain, agitation, or anxiety increases the risk of physiological instability and mobilisation-related adverse events and should be addressed before activity initiation [57]. Importantly, lack of cooperation or transient refusal should not be interpreted as permanent ineligibility; reassessment following optimisation of analgesia, sedation, or delirium management frequently allows progression of rehabilitation as the clinical status evolves [55].

Physiological Safety Considerations

Physiological thresholds commonly used to guide safe initiation and termination of mobilisation in critically ill patients are summarised in Table 2.

**Table 2:** Physiological safety criteria for ICU mobilisation HR: heart rate; SBP: systolic blood pressure; MAP: mean arterial pressure; RR: respiratory rate; SpO2: oxygen saturation; FiO2:  fraction of inspired oxygen; PEEP: positive end-expiratory pressure.

System	Starting criteria	Stopping criteria
Cardiovascular [36,58,59]	HR 60–130 bpm; SBP 90–180 mmHg or MAP 60–100 mmHg	HR <60 or >130 bpm; SBP <90 or >180 mmHg; MAP <60 or >100 mmHg
Respiratory [11,60]	RR 5–40 breaths/min; SpO₂ ≥88%; FiO₂ <0.6; PEEP <10 cmH₂O; airway secured	RR <5 or >40; SpO₂ <88%; risk of airway dislodgement; severe ventilator asynchrony
Neurological [61,62]	Able to open eyes to voice; cooperative or lightly sedated	Reduced consciousness; agitation; new neurological deterioration
Other [63]	No active bleeding or unstable fractures	New arrhythmia; chest pain; syncope; falls; device removal; patient intolerance

ICU-AW management protocol: an algorithmic approach

**Figure 2:**
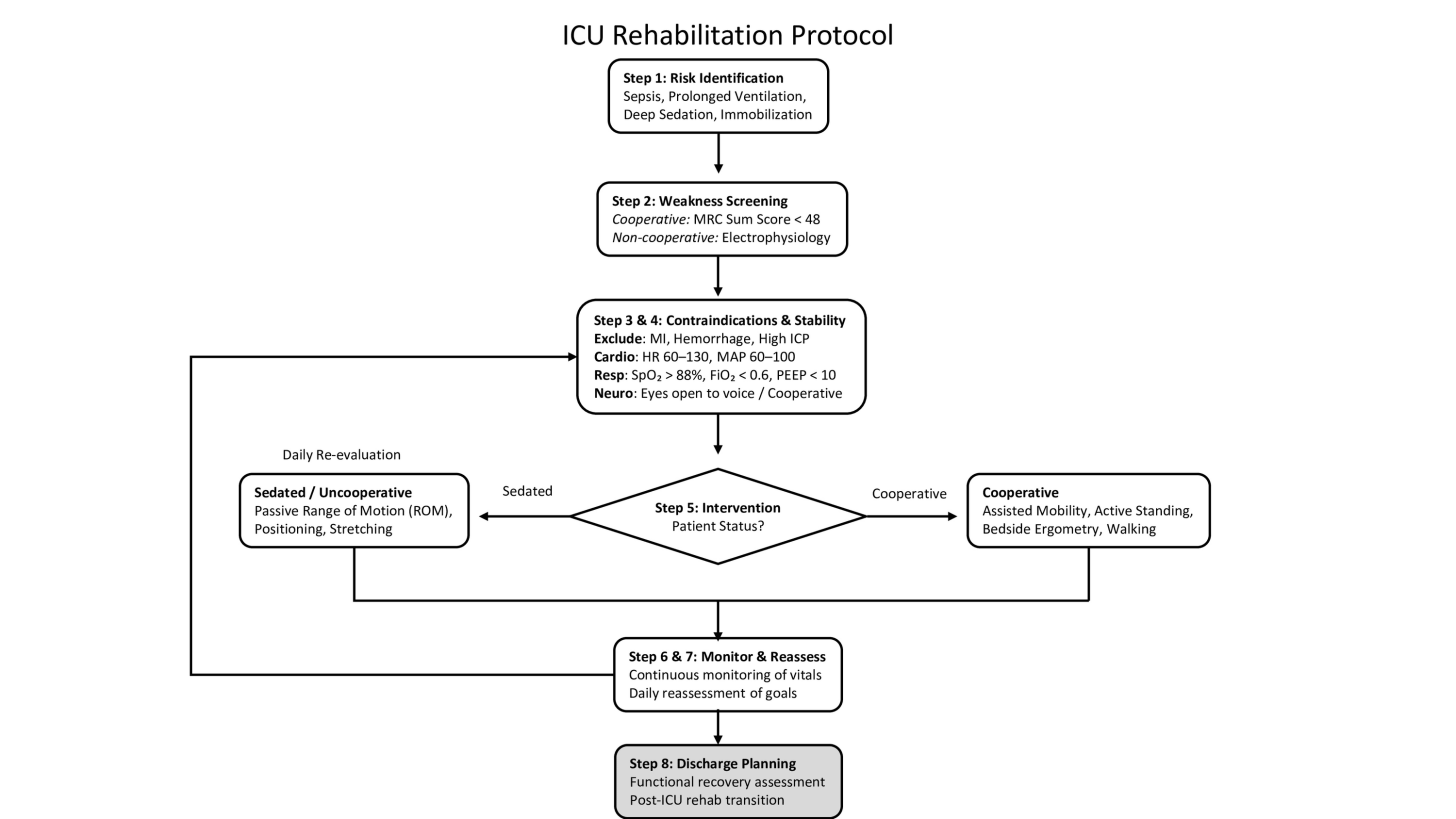
ICU-acquired weakness (ICU-AW) rehabilitation algorithmic protocol This original figure outlines a staged rehabilitation progression based on patient consciousness, physiological stability, and neuromuscular function [45,46,59,61]. MRC: Medical Research Council; MI: myocardial infarction; ICP: intracranial pressure; HR: heart rate; MAP: mean arterial pressure; PEEP: positive end-expiratory pressure. Image credits: Created by Dr. Anurag Tripathi, using Microsoft PowerPoint (Microsoft Corp., Redmond, WA, USA).

A pragmatic, stage-based algorithm to guide rehabilitation progression in ICU-acquired neuromuscular weakness is shown in Figure 2.

Effects of rehabilitation in critically ill patients

Critically ill patients who develop ICU-AW secondary to CIM or CIP frequently experience persistent functional limitations, reduced independence in activities of daily living, and impaired health-related quality of life. Despite the clinical burden of these conditions, a Cochrane systematic review identified no randomised controlled trials specifically evaluating rehabilitation interventions in patients with established CIM or CIP, highlighting a substantial evidence gap in this population [37].

Observational data nevertheless suggest that structured rehabilitation may confer functional benefits. In a cohort of patients with CIP undergoing inpatient rehabilitation, significant gains were observed in lower-limb muscle strength, Functional Independence Measure (FIM) scores, and walking ability following a mean rehabilitation duration of 38 ± 19 days. Earlier transfer to rehabilitation was associated with greater improvement in motor FIM scores, and clinically meaningful gains in activities such as transfers, dressing, and ambulation were achieved in the majority of patients [38].

Evidence derived from ICU-based rehabilitation studies further supports the potential value of early mobilisation strategies. A meta-analysis of five studies involving 120 patients demonstrated improved muscle strength at ICU discharge, reflected by a pooled increase in MRC sum score and a higher likelihood of independent ambulation at hospital discharge [64]. In a larger meta-analysis encompassing 60 trials and over 5,000 patients, early physical rehabilitation was associated with modest improvements in physical function at discharge and reductions in ICU and hospital length of stay. However, consistent effects on mortality or long-term quality of life were not observed, and task-specific, higher-intensity interventions appeared to yield greater benefits than predominantly passive approaches [65].

In deeply sedated and mechanically ventilated patients, the effects of passive rehabilitation appear limited. A systematic review reported attenuation of muscle thickness loss, improved microcirculatory parameters, and favourable short-term inflammatory responses, but did not demonstrate a reduction in the incidence of ICU-AW [66]. From a broader perspective, no disease-specific therapeutic interventions currently exist for CIM or CIP, and management remains focused on preventive strategies such as early mobilisation, optimisation of nutritional support, and minimisation of exposure to neuromuscular blocking agents and corticosteroids [67].

Overall, current evidence suggests that early rehabilitation improves short-term muscle strength, mobility, and healthcare efficiency in critically ill patients, while its impact on mortality and long-term functional outcomes remains inconsistent. Well-designed randomised controlled trials specifically targeting patients with CIM and CIP are urgently needed to define optimal rehabilitation strategies, determine appropriate intervention intensity and timing, and clarify long-term benefits.

Barriers to rehabilitation in the ICU

Despite increasing evidence supporting early rehabilitation in critically ill patients, its integration into routine ICU practice remains inconsistent. Point-prevalence studies have demonstrated low utilisation of rehabilitation services and limited mobilisation activities, even among patients who are clinically stable and meet eligibility criteria for mobilisation [68,69].

System-Level Barriers

At the healthcare system level, barriers include inadequate staffing ratios, competing clinical priorities, limited time availability, insufficient training, and restricted access to specialised equipment required for safe mobilisation [70]. In many ICUs, rehabilitation activities compete with other high-acuity tasks, and the absence of standardised mobilisation pathways, along with unclear role delineation within multidisciplinary teams, further hampers consistent implementation. In low- and middle-income country (LMIC) settings, these challenges are further compounded by budgetary constraints, competing healthcare priorities, and limited cost-effectiveness data for ICU rehabilitation programmes, which may restrict investment in dedicated rehabilitation services.

Provider-Level Barriers

Provider-related factors also contribute substantially. Concerns regarding patient safety, haemodynamic instability, and exercise tolerance frequently delay or prevent mobilisation, particularly in patients receiving vasoactive support or mechanical ventilation. Notably, most studies examining provider perceptions have focused on physicians and physiotherapists, with limited representation of nursing staff and minimal inclusion of respiratory therapists, despite their central role in daily ICU care and mobilisation decision-making [69].

Patient-Related Barriers

Patient-specific factors further restrict participation in rehabilitation. Severe muscle weakness, delirium, ongoing organ dysfunction, fatigue, and psychological distress commonly limit tolerance of mobilisation interventions. In addition, excessive or prolonged sedation remains a major modifiable obstacle in many ICUs, reducing patient engagement and reinforcing clinician reluctance to initiate rehabilitation activities [70].

Collectively, these system-, provider-, and patient-level barriers continue to limit the consistent delivery of rehabilitation interventions in routine ICU practice, even in settings where clinical stability would permit mobilisation.

Future research priorities

Future research in ICU-acquired neuromuscular weakness should move beyond heterogeneous ICU populations and protocol-driven mobilisation strategies toward more targeted, mechanistic, and implementation-focused approaches. Priorities include conducting disease-specific randomised controlled trials in patients with established CIM and CIP rather than mixed critical care cohorts, and undertaking dose-response studies to define the optimal timing, intensity, and progression of rehabilitation interventions across different phases of critical illness and recovery. There is also a need to develop biomarker- and phenotype-guided rehabilitation strategies by integrating tools such as muscle ultrasound, electrophysiology, and inflammatory or metabolic markers to enable personalised intervention selection. In addition, implementation and health-systems research in resource-limited settings is essential to establish scalable, multidisciplinary rehabilitation models that can be integrated into routine ICU practice in LMICs.

## Conclusions

CIM and CIP should be recognised not merely as complications of prolonged intensive care, but as modifiable determinants of ventilator dependence, functional disability, and long-term recovery. While early rehabilitation has become an accepted component of ICU care, its delivery remains inconsistent, and benefits are maximised only when interventions are timed, targeted, and integrated within multidisciplinary practice. Current evidence supports a shift away from uniform, protocol-driven mobilisation toward individualised, stage-specific rehabilitation strategies that account for illness severity, physiological reserve, and evolving recovery trajectories. Adjunctive modalities such as neuromuscular electrical stimulation and respiratory muscle training should be viewed as complementary tools rather than substitutes for active rehabilitation.

From a clinical perspective, rehabilitation in ICU-AW should be approached with the same deliberation as ventilation or sedation management, initiated early when feasible, titrated to patient tolerance, and reassessed daily. Embedding rehabilitation as a core therapeutic intervention, rather than an optional add-on, represents the most immediate and actionable step to improve outcomes for survivors of critical illness. Effective implementation of rehabilitation in ICU-AW ultimately depends on a coordinated interdisciplinary approach involving physicians, nurses, physiotherapists, and respiratory therapists, ensuring safe, consistent, and patient-centred delivery of care across the ICU-to-recovery continuum.

## Appendices

**Table 3:** SANRA-based quality assessment of the narrative review Assessment of methodological and reporting quality of the narrative review using the SANRA (Scale for the Assessment of Narrative Review Articles) criteria [12], with item-wise scoring and justification.

Item	Score	Justification
Importance	2	Clearly established clinical burden
Aim	2	Explicitly stated
Literature search	2	Structured and transparent
Referencing	2	Comprehensive and current
Scientific reasoning	1	Good synthesis but partially descriptive
Presentation	1	Minor redundancy in some sections
